# Molecular Mechanisms for the Coupling of Endocytosis to Exocytosis in Neurons

**DOI:** 10.3389/fnmol.2017.00047

**Published:** 2017-03-13

**Authors:** Zhenli Xie, Jiangang Long, Jiankang Liu, Zuying Chai, Xinjiang Kang, Changhe Wang

**Affiliations:** ^1^Center for Mitochondrial Biology and Medicine, The Key Laboratory of Biomedical Information Engineering of Ministry of Education, School of Life Science and Technology, Xi’an Jiaotong UniversityXi’an, China; ^2^Frontier Institute of Science and Technology, Xi’an Jiaotong UniversityXi’an, China; ^3^State Key Laboratory of Membrane Biology, Peking UniversityBeijing, China; ^4^Beijing Key Laboratory of Cardiometabolic Molecular Medicine, Institute of Molecular Medicine, Peking UniversityBeijing, China; ^5^College of Life Sciences, Liaocheng UniversityLiaocheng, China; ^6^Key Laboratory of Medical Electrophysiology, Ministry of Education of China, Collaborative Innovation Center for Prevention and Treatment of Cardiovascular Disease, Institute of Cardiovascular Research, Southwest Medical UniversityLuzhou, China

**Keywords:** exocytosis, endocytosis, vesicle recycling, calmodulin, synaptotagmin, SNARE

## Abstract

Neuronal communication and brain function mainly depend on the fundamental biological events of neurotransmission, including the exocytosis of presynaptic vesicles (SVs) for neurotransmitter release and the subsequent endocytosis for SV retrieval. Neurotransmitters are released through the Ca^2+^- and SNARE-dependent fusion of SVs with the presynaptic plasma membrane. Following exocytosis, endocytosis occurs immediately to retrieve SV membrane and fusion machinery for local recycling and thus maintain the homeostasis of synaptic structure and sustained neurotransmission. Apart from the general endocytic machinery, recent studies have also revealed the involvement of SNARE proteins (synaptobrevin, SNAP25 and syntaxin), synaptophysin, Ca^2+^/calmodulin, and members of the synaptotagmin protein family (Syt1, Syt4, Syt7 and Syt11) in the balance and tight coupling of exo-endocytosis in neurons. Here, we provide an overview of recent progress in understanding how these neuron-specific adaptors coordinate to ensure precise and efficient endocytosis during neurotransmission.

Neurotransmission based on the exocytosis of synaptic vesicles (SVs) and the subsequent SV membrane retrieval through endocytosis are crucial for efficient neuronal communication, the integrity of neuronal circuits, and normal brain function (Chapman, 2002; Sudhof, 2004; Wu L. G. et al., 2014). With the arrival of an action potential, extra-synaptic Ca^2+^ flows into the nerve terminals and triggers soluble N-ethylmaleimide-sensitive factor attachment protein receptor (SNARE) protein-dependent vesicle exocytosis (Südhof and Rothman, 2009; Jahn and Fasshauer, 2012; Rizo and Xu, 2015). The released neurotransmitters diffuse across the synaptic cleft and activate or inhibit the postsynaptic compartment. After exocytosis, fused SV components are locally retrieved from the neuronal surface through endocytosis, which is tightly coupled to exocytosis. Precise and efficient endocytosis is critical for the preservation of presynaptic morphology and structural integrity, the replenishment of presynaptic vesicle pools, and sustained neurotransmission during continuous neuronal activity (Saheki and De Camilli, 2012; Wu L. G. et al., 2014; Leitz and Kavalali, 2016).

Several modes of endocytosis operate to ensure a sufficient and precise vesicle-recycling rate during neurotransmission. Clathrin-mediated endocytosis (CME), the best-characterized endocytic pathway, is known to be the predominant route of vesicle retrieval with slow kinetics (time constant: 10–30 s) following exocytosis (Granseth et al., 2006; Jung and Haucke, 2007; McMahon and Boucrot, 2011). The elevated neuronal activity also elicits bulk endocytosis, which internalizes a large area of plasma membrane, forms an endosome-like endocytic structure, and is finally converted into releasable SVs by a mechanism that remains elusive (Clayton et al., 2008; Smith et al., 2008; Saheki and De Camilli, 2012; Wu L. G. et al., 2014). The kiss-and-run mode of exo-endocytosis probably represents the fast component of SV endocytosis, during which SVs release their contents through a transient nanometer-sized fusion pore and are retrieved rapidly without full collapse into the plasma membrane (He and Wu, 2007; Rizzoli and Jahn, 2007; Alabi and Tsien, 2013). In addition, ultrafast endocytosis has been revealed by electron microscopic analysis (Watanabe et al., 2013) and membrane capacitance (Cm) recordings (Wu et al., 2009; Mahapatra et al., 2016), which are not discussed in detail here because of uncertainty about the nature of these endocytic pathways.

## Exo-Endocytosis Coupling

Although endocytosis is predominantly a constitutive process in most non-neuronal cells, SV endocytosis is primarily an activity-dependent form of membrane retrieval that is spatiotemporally coupled to exocytosis. Upon depolarization, docked vesicles diminish while clathrin-coated pits and structures associated with bulk endocytosis increase near the release sites (Gad et al., 1998; Gundelfinger et al., 2003; Hosoi et al., 2009; Wang et al., 2016), representing exocytosis and the tightly-coupled endocytosis. Consistently, Cm recordings have revealed endocytosis as a stimulation-dependent form of membrane retrieval, in which exocytosis is represented as a Cm jump upon depolarization and the subsequent Cm decay indicates the process of compensatory endocytosis (Zhang et al., 2004; Wu and Wu, 2007; Yamashita et al., 2010). Importantly, the Cm traces reliably decay back to baseline within seconds to minutes after exocytosis, indicating that endocytosis retrieves an amount similar to that of exocytosed SV membrane (Lou et al., 2008; Yamashita et al., 2010; Wang et al., 2016). Furthermore, blockade of exocytosis by cleaving SNARE proteins with botulinum neurotoxins also abolishes endocytosis (Wu et al., 2005; Yamashita et al., 2005), implying a critical role of exocytosis in the initiation of endocytosis. Given the limitation of Cm recordings in small conventional synapses, the optical imaging of fluorescent dyes such as FM1–43, or dextran uptake, has permitted studies of vesicle recycling in neuronal terminals (Virmani et al., 2003; Deák et al., 2004; Clayton et al., 2010; Wang et al., 2016). Tagging vesicular proteins with pHluorin, a pH-sensitive green fluorescent protein that allows the direct visualization of exocytosis and the subsequent endocytosis in living nerve terminals, has also confirmed the tight coupling of synaptic endocytosis to exocytosis in terms of both timing and amount (Poskanzer et al., 2003; Ferguson et al., 2007; Hua et al., 2011; Yao et al., 2011).

## Ca^2+^/Calmodulin in Exo-Endocytosis Coupling

Although there is extensive evidence that Ca^2+^ influx plays critical role in compensatory endocytosis, whether and how cytosolic Ca^2+^ regulates exo-endocytosis coupling is rather controversial. Accumulating evidence has shown that a transient elevation in cytosolic Ca^2+^ triggers and accelerates both clathrin-dependent and clathrin-independent endocytosis in neurons and neuroendocrine cells (Balaji et al., 2008; Hosoi et al., 2009; Sun et al., 2010; Leitz and Kavalali, 2016). However, the Ca^2+^-dependence of exo-endocytosis is diverse among different preparations (Wu and Wu, 2014; Wu L. G. et al., 2014). Endocytosis can also occur independent of cytosolic Ca^2+^ (Ryan et al., 1993; Granseth et al., 2006), and increasing the intracellular Ca^2+^ concentration slows exo-endocytosis in many cases (von Gersdorff and Matthews, 1994; Leitz and Kavalali, 2011; Armbruster et al., 2013). Nonetheless, the critical roles of cytosolic Ca^2+^ in SV exocytosis make it inconclusive whether Ca^2+^ influx directly mediates exo-endocytosis coupling and thus controls the timing and amount of compensatory endocytosis independent of exocytosis, although great efforts have been made to dissect this by manipulating exocytosis (Sun et al., 2002; Wu et al., 2009; Yao et al., 2011). Thus, the exact role of Ca^2+^ in the coupling of SV exo-endocytosis remains a pending question and needs more thorough investigations.

Several endocytic Ca^2+^ sensors and effectors have been shown to initiate and mediate Ca^2+^-dependent endocytosis, in which calmodulin is involved in most forms of endocytosis and synaptotagmin is a dual Ca^2+^ sensor for both exocytosis and endocytosis. Calcineurin functions as a key mediator of Ca^2+^/calmodulin in exo-endocytosis by dephosphorylating endocytic proteins known as dephosphins (Cousin and Robinson, 2001; Saheki and De Camilli, 2012). Typically, many proteins involved in different stages of CME (e.g., dynamin, synaptojanin, amphiphysin, epsin and Eps15) are constitutively phosphorylated as an inactive conformation in resting nerve terminals (Liu et al., 1994; Chen et al., 1999; Lee et al., 2004, 2005). During synaptic activity, these dephosphins undergo rapid dephosphorylation by the Ca^2+^/calmodulin-activated calcineurin to drive endocytosis *via* their enhanced binding to other endocytic factors or by dephosphorylation-dependent activation (Liu et al., 1994; Slepnev et al., 1998; Anggono et al., 2006; Saheki and De Camilli, 2012). The regulation of CME by calcineurin has been confirmed by the inhibition of slow endocytosis with calcineurin blockers, or the knockdown/knockout of calcineurin (Engisch and Nowycky, 1998; Sun et al., 2010; Armbruster et al., 2013; Wu X. S. et al., 2014). In addition, calcineurin also mediates bulk endocytosis by dephosphorylating dynamin 1 during elevated neuronal activity (Clayton et al., 2009, 2010). It has been proposed that the GTPase activity of dynamin is essential for vesicle fission during CME, bulk endocytosis and kiss-and-run, while its phosphorylation-dephosphorylation cycle is also critical for activity-dependent bulk endocytosis (Marks et al., 2001; Yamashita et al., 2005; Anggono et al., 2006; Clayton and Cousin, 2009; Anantharam et al., 2011). However, the dynamin-dependency of bulk endocytosis remains controversial because it still occurs robustly in the absence of dynamin 1, which might be due to the compensatory effect of other dynamin isoforms (Hayashi et al., 2008; Raimondi et al., 2011; Lou et al., 2012; Fan et al., 2016). Finally, dynamin and the calcineurin-dependent dynamin-syndapin interaction have also been demonstrated to regulate the kiss-and-run mode of exo-endocytosis and the quantal size of neurotransmitter release by limiting the fusion pore dilation under elevated stimulation (Graham et al., 2002; Samasilp et al., 2012).

In addition to calcineurin, myosin light-chain kinase is another co-effector functioning to accelerate both the slow and fast forms of exo-endocytosis through the activity-dependent phosphorylation of myosin at the downstream of Ca^2+^/calmodulin (Yue and Xu, 2014; Li et al., 2016). A recent study has also defined critical roles of calmodulin in regulating the intrinsic membrane-remodeling activity *via* a Ca^2+^-dependent interaction with Rvs167 in yeast and several endocytic N-BAR domain proteins such as endophilins and amphiphysins in mammalian cells (Myers et al., 2016).

## Synaptotagmin Proteins in Exo-Endocytosis Coupling

Synaptotagmins (Syts), a family of type I membrane proteins with evolutionarily conserved cytosolic tandem C_2_ domains (C_2_A and C_2_B), are well-characterized Ca^2+^ sensors that initiate SNARE-dependent vesicle fusion during synaptic transmission and hormone secretion (Chapman, 2002; Gustavsson and Han, 2009; Südhof and Rothman, 2009; Pang and Südhof, 2010). At least 17 mammalian Syt isoforms have been identified, the detailed characterizations of which are summarized in recent reviews (Gustavsson and Han, 2009; Pang and Südhof, 2010). All Syt members bind the clathrin-adaptor protein AP-2 with high affinity (*K*_d_ = 0.1–1.0 nM) and some Syts have been shown to function in different endocytic pathways (Zhang et al., 1994; Li et al., 1995; Chapman et al., 1998; Yao et al., 2011). Syt1, the prototypical Syt protein functioning as the primary Ca^2+^ sensor for exocytosis, has also been proposed to be a major Ca^2+^-sensing protein that promotes CME upon exocytosis (Haucke et al., 2000; Jarousse and Kelly, 2001; Poskanzer et al., 2003). Cm recordings, electron microscopy, FM uptake and pHluorin assays have reliably revealed dramatic endocytic defects in Syt1-deficient cells from a variety of organisms (Poskanzer et al., 2003; Nicholson-Tomishima and Ryan, 2004; Yao et al., 2011, 2012). Meanwhile, Syt1 has also been demonstrated to bind the μ2 subunit of the endocytic adaptor protein AP-2 and the μ-homology domain of stonin-2 through its C2B domain (Zhang et al., 1994; Haucke et al., 2000; Jarousse and Kelly, 2001; Walther et al., 2001; Kaempf et al., 2015). However, the direct regulation of Syt1 in CME has been challenged due to that the endocytic defects may be secondary to the impaired exocytosis caused by Syt1 deficiency (Poskanzer et al., 2006; Yao et al., 2011). A recent study has provided direct evidence that Syt1 indeed functions as a Ca^2+^ sensor for SV endocytosis by uncoupling the function of Syt1 in exo- and endocytosis in hippocampal neurons (Yao et al., 2011). Then, with cell-attached Cm recordings, another group validated that Syt1 functions to modulate the Ca^2+^-dependence of CME probably by AP-2-dependently prolonging the duration of fission pore closure (Yao et al., 2012).

Syt7 is ubiquitously expressed at early stage of development but is later restricted to dividing cells, neuroendocrine cells, and presynaptic neuronal structures (Virmani et al., 2003). Syt7 binds Ca^2+^ with a high apparent affinity and slow kinetics, and thus mainly functions as a slow Ca^2+^ sensor to mediate the slow phase of exocytosis known as asynchronous release, as well as fusion-pore expansion and synaptic facilitation (Maximov et al., 2008; Schonn et al., 2008; Liu et al., 2014; Neuland et al., 2014; Wu et al., 2015). Interestingly, Syt7 is extensively spliced and exhibits a broad variety of alternative splice variants, among which the short Syt7 variant lacking both of the C2 domains inhibits CME but accelerates exo-endocytosis in response to intense stimulation, while the regular full-length Syt7 directs synaptic endocytosis into a slow-recycling CME (von Poser et al., 2000; Virmani et al., 2003). A recent study also defined Syt7 as a Ca^2+^ sensor for SV replenishment (Liu et al., 2014), confirming the regulatory role of Syt7 in SV recycling. Furthermore, Syt7 also plays a critical role in the occurrence of kiss-and-run probably by mediating the push-and-pull regulation of fusion pore dilation (Segovia et al., 2010; Neuland et al., 2014). It has been proposed that Ca^2+^ binding to the C2A domain of Syt7 is sufficient to trigger fusion-pore opening but the resulting pores are unstable, thus leading to a dramatic increase in kiss-and-run fusion events. In contrast, Ca^2+^ binding to the C2B domain facilitates the continuous expansion of fusion pores, making Syt7 a critical regulator of the Ca^2+^-dependent occurrence of kiss-and-run and full-fusion events (Segovia et al., 2010; Neuland et al., 2014).

Syt4 and Syt11 are classified as non-Ca^2+^-binding Syts because of an aspartate-to-serine substitution in a Ca^2+^-coordination site of the C_2_A domain, and they do not bind Ca^2+^ biochemically (von Poser et al., 1997; Dai et al., 2004; Dean et al., 2009). Syt4 has been reported to regulate fusion-pore and fusion modes in both endocrinal cells and neurons, but the effects fail to reach a consensus in these preparations. Syt4 overexpression favors the occurrence of kiss-and-run and increases the duration of fusion pore dilation in PC12 cells (Wang et al., 2001, 2003; Zhang et al., 2010). Cell-attached Cm recording also revealed prolonged lifetime and smaller downward Cm steps of fission pores during endocytosis (Zhang et al., 2010). In contrast, Syt4 deficiency accelerates the rapid component of endocytosis probably through the enhanced kiss-and-run in the peptidergic nerve terminals of posterior pituitary neurons (Zhang et al., 2009). Similarly, Syt4 inhibits BDNF release in both axons and dendrites but with distinct mechanisms, in which presynaptic Syt4 decreases frequency of spontaneous quantal release while postsynaptic Syt4 limits quantal size by favoring kiss-and-run modes of exo-endocytosis (Dean et al., 2009).

Syt11 is a newly-defined endocytic regulator that inhibits CME and bulk endocytosis in neurons probably through distinct mechanisms (Wang et al., 2016). Disruption of this inhibitory role by Syt11-knockdown induces excessive membrane retrieval, accelerates vesicle pool replenishment, and facilitates sustained neurotransmission, indicating a critical role of Syt11 as a clamp protein to ensure the precise coupling and balance of endocytosis to exocytosis during neurotransmission (Wang et al., 2016). Since Syt11 does not bind Ca^2+^ biochemically, there may also be a Ca^2+^-sensitive inhibitor to ensure the precise Ca^2+^-dependency of exo-endocytosis, especially during sustained neuronal activities.

## SNARE Proteins and Synaptophysin in Exo-Endocytosis Coupling

In addition to Ca^2+^ influx upon depolarization, exocytosis itself is required for the initiation of compensatory SV endocytosis, which is abolished by the cleavage of SNARE proteins essential for exocytosis with botulinum neurotoxins (Hosoi et al., 2009; Xu et al., 2013). A debated issue is that exocytosis-mediated plasma membrane expansion and surface tension reduction may serve to initiate the local membrane curvature (membrane buds) for internalization (Dai et al., 1997; Anantharam et al., 2010; Diz-Muñoz et al., 2013; Hassinger et al., 2017). Meanwhile, the delivery of PI(4,5)P_2_-lacking SV membranes to the plasma membrane makes these budding sites competent for the recruitment of endocytic scaffolding proteins and the formation of coated pits (Wenk and De Camilli, 2004; McMahon and Gallop, 2005; Haucke et al., 2011; Saheki and De Camilli, 2012; Puchkov and Haucke, 2013). Furthermore, some classical exocytic proteins, especially Syts, SNARE proteins and synaptophysin, also function to couple exo-endocytosis.

SNARE proteins are critical for membrane fusion, while recent studies have also implied a significant contribution of synaptobrevins (also termed VAMPs, vesicle-associated membrane proteins), syntaxin, and SNAP-25 in the coupling of SV exo-endocytosis. Synaptobrevin-2 (VAMP2) deficiency impairs the fast component of compensatory endocytosis and the rapid re-use of SVs in hippocampal neurons (Deák et al., 2004), while the cleavage of VAMP2 and VAMP3 with tetanus toxin blocks both the slow and fast modes of endocytosis in nerve terminals of the calyx of Held (Hosoi et al., 2009; Xu et al., 2013). A recent study has also established an essential role of synaptobrevin in slow endocytosis in hippocampal neurons (Zhang et al., 2013). VAMP4 also plays critical roles in activity-dependent bulk endocytosis in hippocampal neurons (Nicholson-Fish et al., 2015). In addition, an early study also revealed the involvement of t-SNARE proteins (syntaxin and SNAP-25 in targeting membrane) in exo-endocytosis coupling in yeast (Gurunathan et al., 2002). Consistently, SNAP25 knockdown inhibits slow SV endocytosis in hippocampal synapses (Zhang et al., 2013), and the cleavage of SNAP-25 with botulinum neurotoxin E impairs both the fast and slow modes of endocytosis in calyx terminals (Xu et al., 2013). Syntaxin 1 clearance with botulinum neurotoxin C also greatly inhibits SV endocytosis at the calyx (Xu et al., 2013), while syntaxin 1A SUMOylation shows a similar inhibitory effect on SV endocytosis in cortical and hippocampal neurons (Craig et al., 2015).

Synaptophysin is the most abundant SV protein; it is exclusively localized to SVs with uncertain roles in SV exocytosis, endocytosis, synapse formation, and other synaptic functions (Janz et al., 1999; Tarsa and Goda, 2002; Takamori et al., 2006). Synaptophysin interacts with dynamin *via* its C-terminal cytoplasmic tail region in a Ca^2+^-dependent manner (Daly et al., 2000; Daly and Ziff, 2002), disruption of which decreases vesicle retrieval and thus neurotransmitter release during intense stimulation, probably due to the impairment of clathrin-independent rapid endocytosis (Daly et al., 2000). A recent study provided direct evidence for the involvement of synaptophysin in exo-endocytosis coupling by using optical imaging of Syt1-pHluorin and SV2-pHluorin. Synaptophysin knockout impairs SV endocytosis during and after sustained neuronal activity, while the C-terminal tail-truncated synaptophysin can only rescue the slow post-stimulus endocytosis (Kwon and Chapman, 2011), indicating the distinct requirement of synaptophysin structural elements in the two phases of exo-endocytosis. These findings validate the critical dual roles of synaptophysin and SNARE proteins in both exocytosis and the exo-endocytosis coupling process; however, which specific endocytic pathways are regulated by these fusion machineries and how these proteins are involved in the compensatory SV endocytosis remain largely elusive.

## Conclusion

**Figure 1:**
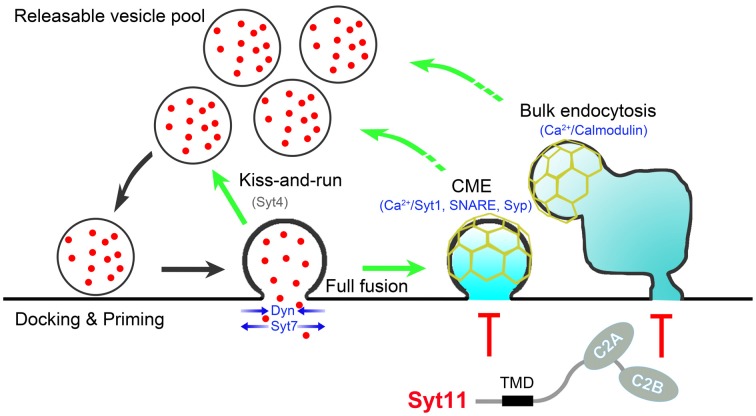
**Schematic presentation showing proteins involved in exo-endocytosis coupling.** During neurotransmission, endocytosis occurs immediately after synaptic vesicle (SV) exocytosis *via* clathrin-mediated endocytosis (CME), kiss-and-run and bulk endocytosis. The Ca^2+^-calmodulin pathway, synaptophysin (Syp) and SNARE proteins, Ca^2+^-binding (Syt1, Syt7) and non-Ca^2+^-binding (Syt4, Syt11) Syts, and other proteins such as dynamin (Dyn) coordinate to control the efficient and precise coupling of SV endocytosis to exocytosis. Syt11 is shown in red due to the nature of negative regulator, and Syt4 is shown in gray since that Syt4 shows different effects on different types fusion-fission events. Scaffolding proteins (e.g., N-BAR/F-BAR/BAR domain-containing proteins (such as FCHo, endophilin, amphiphysin, syndapin), intersectin, Rab3, CDC42, N-WASP, SNX9 and Eps15), downstream effectors (e.g., calcineurin, myosin, CDC2 and CDK5), phosphoinositide metabolism (e.g., PI(4,5)P_2_, cholesterol, synaptojanin, PI 3-kinase and PIPK1γ), and other general endocytic machineries (clathrin, AP-2, AP180, Epsin and stonin) are excluded to simplify the cartoon.

Recent advances paint an extremely complex picture of the tight exo-endocytosis coupling in neurons. At least three different endocytic pathways, CME, activity-dependent bulk endocytosis, and the kiss-and-run mode of fast endocytosis, cooperate to couple SV endocytosis to exocytosis with different neuronal activities. The Ca^2+^-calmodulin-calcineurin pathway, synaptophysin and SNARE proteins, Ca^2+^-binding Syt members, and other positive regulators work together with endocytic inhibitors such as non-Ca^2+^-binding Syts to provide a fine-tuning mechanism for the efficient and precise coupling of SV endocytosis to exocytosis (Figure 1). Membrane lipid structures and proteins involved in phosphoinositide metabolism also play critical roles in the exo-endocytosis coupling. In addition, scaffolding and effector proteins essential for non-neuronal endocytosis are also necessary for exo-endocytosis coupling in neurons. However, uncertainty about the functions of these endocytic regulators and the co-existence of several other endocytic pathways with distinct kinetics and molecular mediators require a more thorough investigation (Wu et al., 2009; Watanabe et al., 2013; Kononenko and Haucke, 2015). Further studies have been challenged due to the limitation of electrophysiological recordings and live fluorescence imaging assays of single-SV recycling in small nerve terminals. Advances in super-resolution microscopy and correlative light and electron microscopy offer new opportunities in this field. In addition, optogenetic stimulation, two-photon imaging, and acute molecular manipulation *in vivo* allow a deep functional analysis of the endocytic regulators that associate SV recycling with brain disorders such as Alzheimer disease, Parkinson disease and emotional disorders.

## Author Contributions

ZX drafted the manuscript with help from JLo, JLi, ZC, XK and CW. All authors coordinated, revised and approved the manuscript.

## Funding

This work was supported by the National Natural Science Foundation of China (31400708, 81571235 and 31670843), the Natural Science Foundation of Heilongjiang Province of China (C201453), the Natural Science Foundation of Shandong Province of China (ZR2016CM16) and the Scientific Research Fund of Heilongjiang Provincial Education Department (12531750 and 12531746). XK was supported in part by the start-up funding of Liaocheng University (318051525).

## Conflict of Interest Statement

The authors declare that the research was conducted in the absence of any commercial or financial relationships that could be construed as a potential conflict of interest.
